# Fractional flow reserve-guided complete vs. culprit-only revascularization in ST-elevation myocardial infarction patients with multivessel disease: a meta-analysis

**DOI:** 10.3389/fcvm.2025.1509912

**Published:** 2025-03-11

**Authors:** Jingxian Yang, Peng Wang, Jun Wan, Na Li, Jiajia Didi, Binger Shen, Xinyu Yang, Feina Li, Yu Zhang

**Affiliations:** ^1^ Center for Evidence-based Medicine, Affiliated Hospital of Chengdu University, Chengdu, Sichuan, China; ^2^ Department of Critical Care Medicine, Affiliated Hospital of Chengdu University, Chengdu, Sichuan, China

**Keywords:** fractional flow reserve, ST-elevation myocardial infarction, multivessel, revascularization, myocardial infarction

## Abstract

**Background:**

Among patients with ST-elevation myocardial infarction (STEMI) and multivessel disease, whether fractional flow reserve (FFR) guided complete revascularization (CR) is superior to the now widely used culprit-only (COR) revascularization is unclear.

**Methods:**

We conducted a search of PubMed, Embase, the Cochrane Library, and CNKI for randomized controlled trials comparing FFR-guided CR with COR in STEMI patients with multivessel disease. Data extraction and analysis adhered to Cochrane guidelines, with major adverse cardiac events as the primary outcome.

**Results:**

This meta-analysis included 6 trials involving 3,482 patients. FFR-guided CR was associated with a reduction in major adverse cardiac events (RR: 0.66, 95% CI: 0.46–0.94, 95% PI: 0.20–2.19), ischemia-driven revascularization (RR: 0.27, 95% CI: 0.19–0.40, 95% PI: 0.16–0.46), and repeat percutaneous coronary interventions (RR: 0.35, 95% CI: 0.22–0.50, 95% PI: 0.16–0.78) compared to COR. However, no difference was observed in all-cause mortality (RR: 1.12, 95% CI: 0.86–1.46, 95% PI: 0.79–1.58) or safety outcomes.

**Conclusion:**

FFR-guided CR reduces major adverse cardiac events compared to COR, though benefits may vary across settings. It significantly lowers ischemia-driven revascularization and repeat percutaneous coronary interventions, with no difference in all-cause mortality compared to COR.

**Systematic Review Registration:**

https://www.crd.york.ac.uk/PROSPERO/view/CRD42024567524, PROSPERO (CRD42024567524).

## Introduction

About 50% of patients with ST-segment elevation myocardial infarction (STEMI) show significant stenosis in more than one non-culprit vessel during coronary angiography (1). These patients typically have a worse prognosis than those without non-culprit lesions (2). Hence, investigating optimal revascularization strategies for STEMI patients with multivessel disease is crucial.

Both US (3) and European guidelines (4) advocate for non-culprit vessel intervention in these patients, based on studies (5, 6) indicating reductions in major adverse cardiovascular events and repeat revascularizations, though not in all-cause or cardiovascular mortality. Angiography alone may not accurately assess non-culprit lesions, as it can either overestimate or underestimate their significance (7).

Fractional flow reserve (FFR) offers a functional assessment of non-culprit lesions through pressure wire measurements, potentially improving angioplasty decisions (8). Recently, a new trial (9) with a largest sample on this topic suggests that FFR-guided complete revascularization(CR) may not reduce major adverse cardiac events more than culprit-lesion-only percutaneous coronary intervention. This systematic review and meta-analysis aim to evaluate whether FFR-guided CR improves outcomes such as major adverse cardiac events and all-cause mortality in STEMI patients with multivessel disease.

## Methods

This meta-analysis adhered to PRISMA guidelines (10) and the Cochrane Handbook for Systematic Reviews of Interventions, (11) registered under PROSPERO (CRD42024567524).

### Search strategy

We searched PubMed, Embase, the Cochrane Library, and CNKI up to July 2024 using keywords related to “STEMI”, “FFR”, “revascularization” and “randomized controlled trials”. Exact search strategies were listed in the Supplementary Table S1. Additional studies were identified by reviewing the reference lists of included articles.

### Study selection

Two reviewers(XY and FL) independently screened titles and abstracts against inclusion criteria, resolving disagreements by consensus or a third reviewer. Inclusion criteria were: (1) randomized controlled trials, (2) comparison of FFR-guided CR with culprit-only revascularization(COR) percutaneous coronary intervention, and (3) STEMI patients with multivessel disease.

### Data extraction

Two authors independently collected information from each included eligible study. The recorded information covering the first author, the total number of features and subjects, follow-up duration, mean age, gender, smoking, diabetes mellitus, hypertension, three-vessel disease and Killip class II–III. A third researcher made the ultimate determination if there were any controversies between the two researchers.

### Outcomes

Primary outcome included major adverse cardiac events, while secondary outcomes covered all-cause mortality, ischemia-driven revascularization, repeat percutaneous coronary intervention, and safety events such as cardiac death and stroke.

### Risk-of-bias assessment

Risk of bias was evaluated using the Cochrane risk of bias tool, categorizing studies into low risk, some concerns, or high risk based on five key domains (12).

### Quality of evidence

Evidence quality was assessed using the Grading of Recommendations, Assessment, Development, and Evaluations approach, initially rated high but downgraded based on limitations, indirectness, inconsistency, imprecision, and other factors (Supplementary Table S4) (13).

### Statistical analysis

The information was examined with Review Manager (Version 5.4) and STATA. Relative risks (RR) and 95% confidence intervals (CI) were calculated, with heterogeneity assessed by Cochran's *Q* test and Higgins *I*2 statistics. Prediction intervals (PI) were calculated to describe the distribution of true effects around the summary effect (14). We used the DerSimonian-Laird method and random-effects models for all meta-analyses, which are conservative as they consider both within- and between-study variability (15). A sensitivity analysis using the “leave-one-out” method was conducted to evaluate the impact of individual studies on the overall outcome. Publication bias was evaluated through funnel plots and Egger's regression. Trial sequential analysis (TSA) (16, 17). was conducted to ensure robustness and to assess the cumulative evidence.

## Results

**Figure 1:**
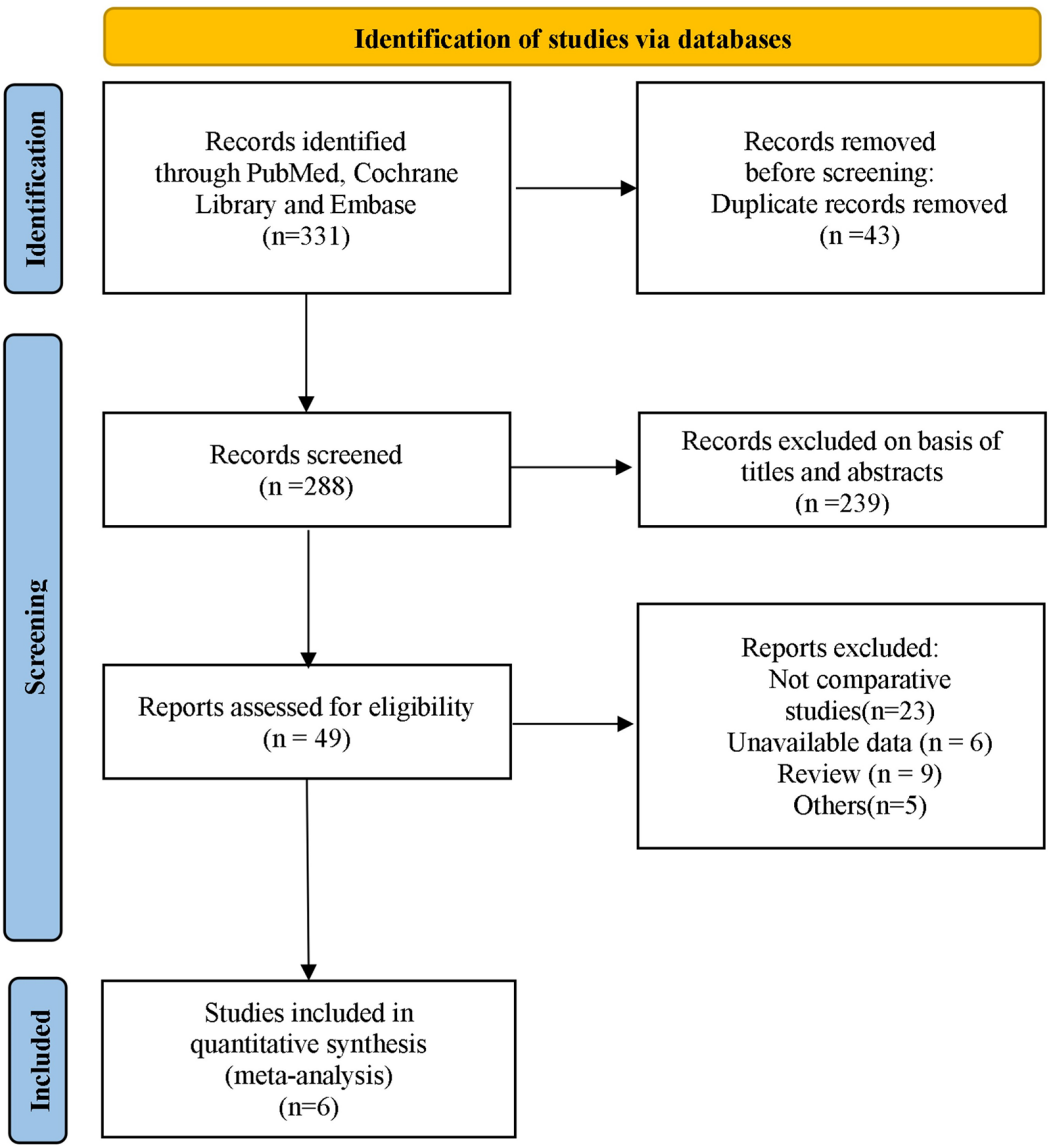
Identification of eligible articles.

A total of 331 articles from the primary literature were found in the databases of PubMed, Embase, the Cochrane Library, and CNKI. Initially, 12 duplicates were identified and promptly eliminated. Following a rigorous screening process of titles and abstracts, a further 258 records were excluded as they did not meet the inclusion criteria. Out of the remaining 61 articles, 19 were discarded because they were not comparative studies, 14 were excluded because they lacked survival information, and 22 were omitted due to insufficient data. In the end, six articles (5, 6, 9, 18–20) were chosen for inclusion in the meta-analysis (Figure 1).

### Eligible study characteristics

**Figure 2:**
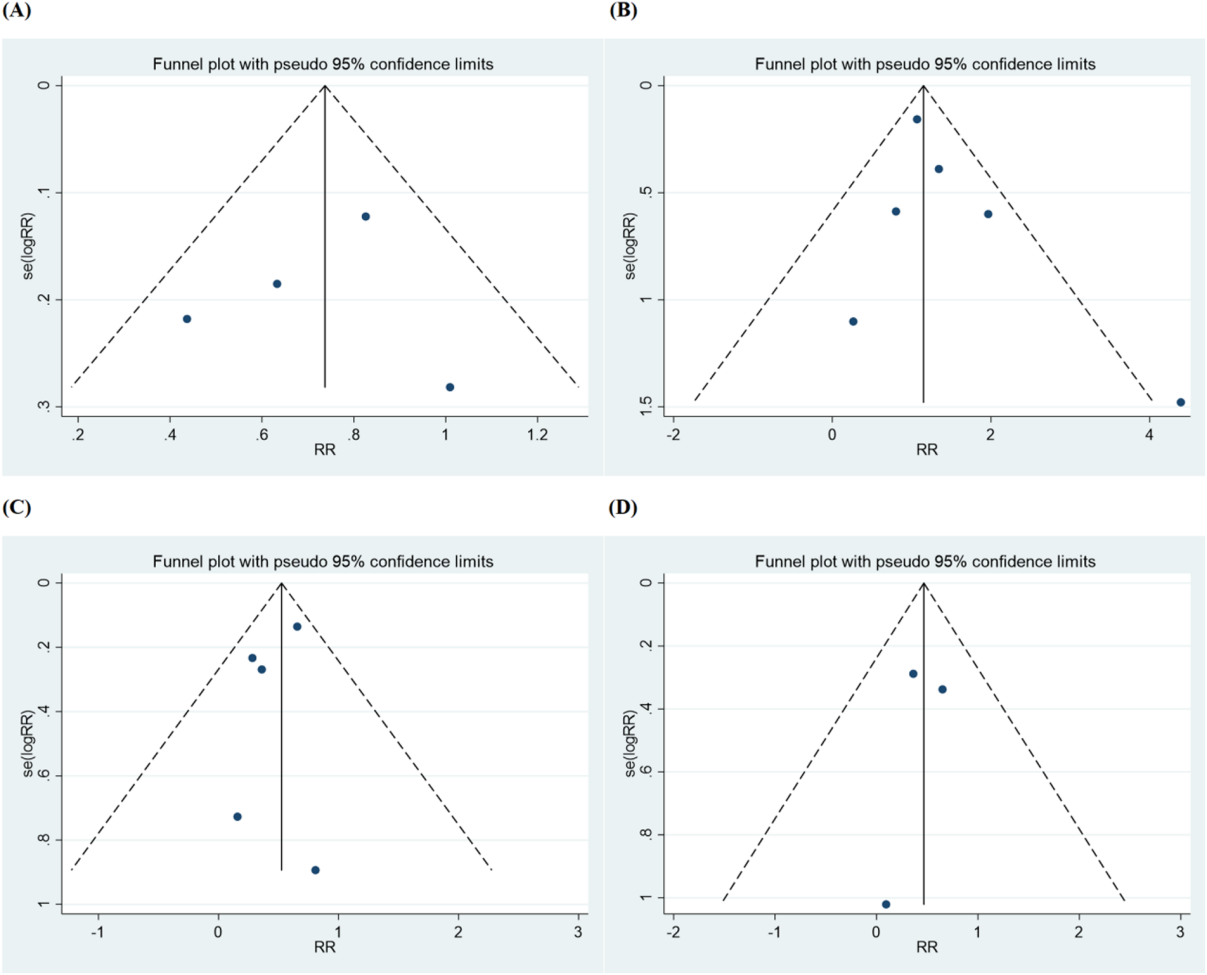
Funnel plot for **(A)** major adverse cardiac event, **(B)** all-cause mortality, **(C)** ischemia-driven revascularization, **(D)** repeat percutaneous coronary intervention.

**Figure 3:**
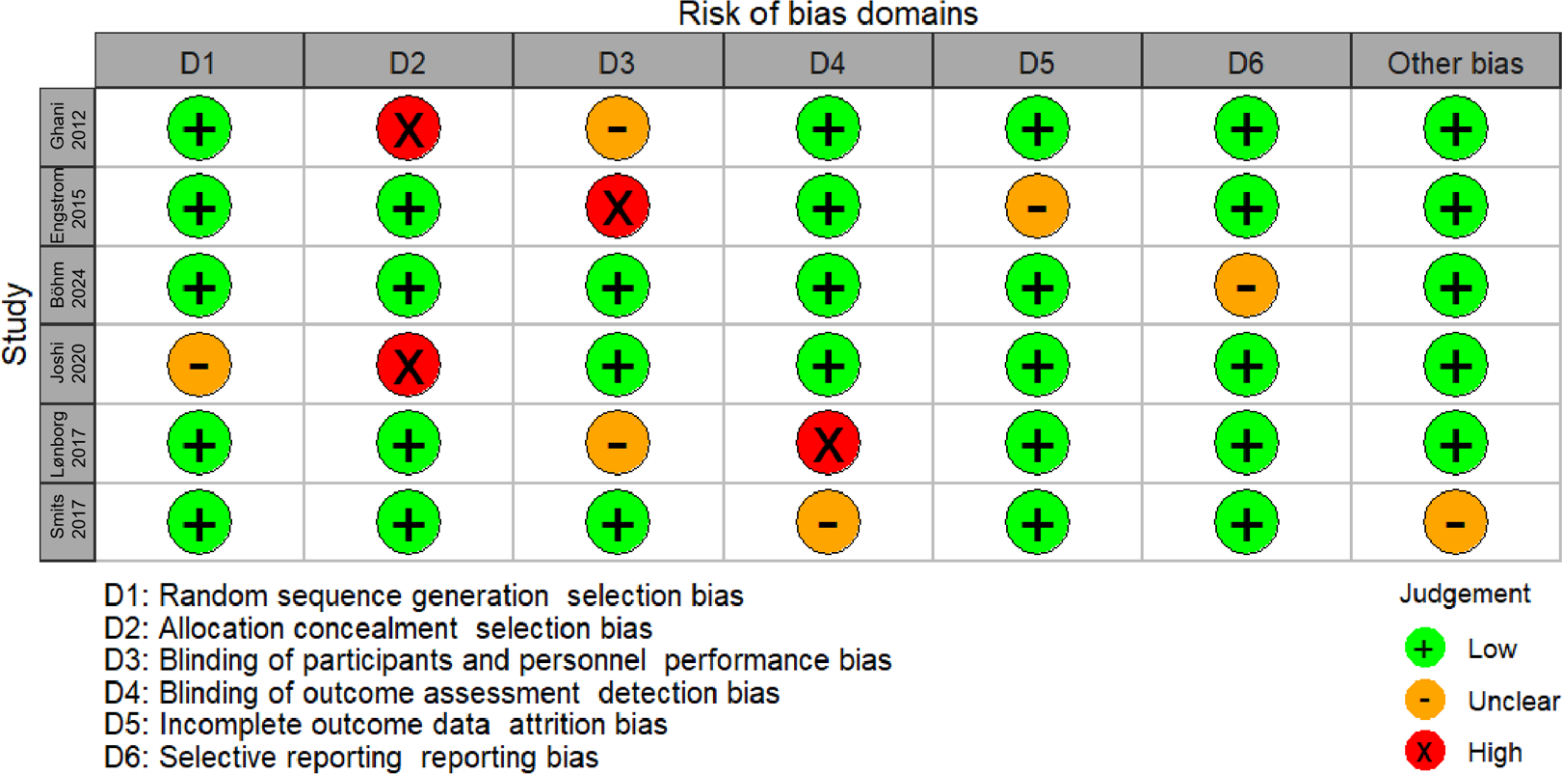
Risk of bias assessment for RCTs (RoB 2.0).

The characteristics of the six articles are summarized in Table 1. These studies were published from 2012–2024. These studies included a total of 3,482 patients, and the number of patients in each study varied from 110–1,542. The summary of six studies is shown in Supplementary Table S2 and the main results extracted from the six articles are shown in Supplementary Table S3. The inverted funnel plots for the primary outcome of major adverse cardiac events and all-cause mortality or ischemia-driven revascularization did not suggest publication bias (Figure 2). The risk of bias assessment for each included trial by domain appear in Figure 3.

**Table 1:** Characteristics of the included randomized controlled trials comparing FFR-guided CR with COR.

Study	Patient	Mean age	Female	Intervention	Indication of non-IRA intervention	Stent type	Killip class II–III
	(N)	(Y)	No. (%)				
Ghani 2012 (5)	121	61.5	24 (29)	FFR-guided PCI in vessels with significant stenosis (<90%) vs cuipirit-vessel PCI plus PCI of several lesion (>90%)	FFR < 0.75 Or Diameter stenosis > 90%	DES 20%	5/1
						BMS 68%	
Engstrøm 2015 (6)	627	63.5	121 (19)	revascularisation guided by FFR values vs no further invasive treatment after primary PCI of the infarct-related artery only	FFR < 0.80 Or Diameter stenosis > 50%	DES 94% BMS 1%	22/20
Böhm 2024 (9)	1,542	65.5	365 (24)	FFR-guided complete revascularization vs culprit-lesion-only PCI	FFR < 0.80 Or Diameter stenosis between 90 and 99%	DES 95%	34/37
Joshi 2020 (18)	110	80.0	35 (32)	FFR-guided complete revascularization vs infarct-related (culprit) artery-only PCI	FFR < 0.80 Or Diameter stenosis >50%	DES 2%	5/4
Lønborg 2017 (19)	197	62.5	40 (20)	FFR–guided complete revascularization vs infarct-related percutaneous coronary intervention only.	FFR < 0.80 Or Diameter stenosis ≥ 90%.	DES 94%	NA
Smits 2017 (20)	885	61.5	202 (23)	non–infarct-related coronary arteries guided by FFR vs culprit-lesion-only PCI	FFR < 0.80 Or Angiographic stenosis ≥50%	DES 97%	15/30

Values are reported as experimental group Control group. N, number; Y, year; NA, not available; FFR, fractional flow reserve; PCI, percutaneous coronary intervention; DES, drug-eluting stent; BMS, bare mental stent.

### Primary outcome

**Figure 4:**
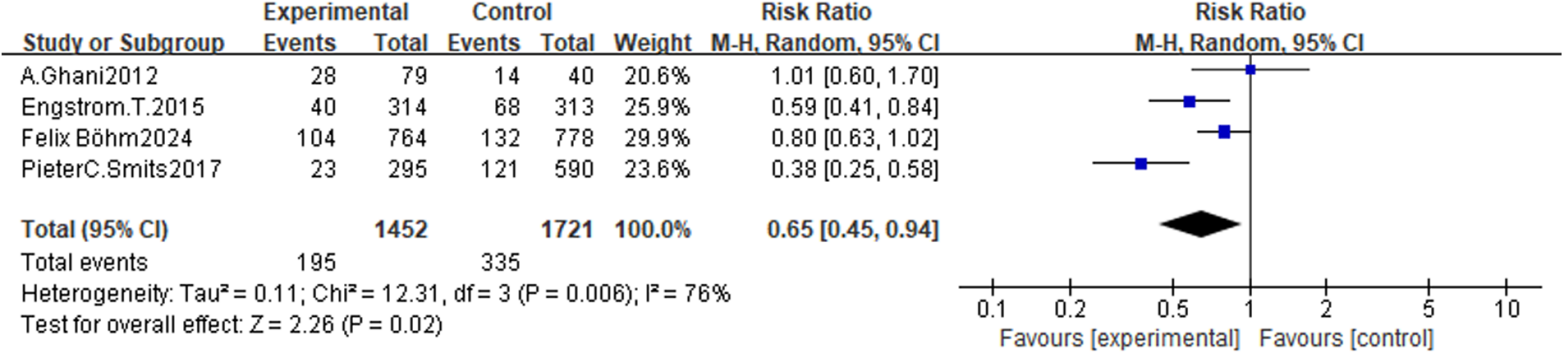
Forest plots for major adverse cardiac events.

Four studies reported major adverse cardiac events (5, 6, 9, 20). Among these studies, 195 events (13.4%) occurred in the 1,452 patients undergoing FFR-guided CR compared with 331 events (19.2%) in 1,721 patients undergoing COR (RR: 0.65, 95% CI: 0.45–0.94, 95% PI: 0.20–2.19; *P* = 0.02) (Figure 4). The 95% CI (0.46–0.94) demonstrates a statistically significant reduction in major adverse cardiac events with FFR-guided CR. However, the 95% PI (0.20–2.19), which includes 1.0, suggests potential variability in the magnitude of this benefit across different populations or settings, consistent with the observed heterogeneity (*I*^2^ = 74%).

Among the trials, moderate significant heterogeneity (*I*^2^ = 74%) was observed, so a leave-one-out sensitivity analysis was conducted to identify potential sources of heterogeneity. Regardless of which study was excluded, there was always a significantly higher risk of major adverse cardiac events in the COR group (Supplementary Table S5). Trial sequential analysis confirmed that the required sample size (2,845) to reach definitive conclusions was achieved (Supplementary Figure S1).

### Secondary outcomes

**Figure 5:**
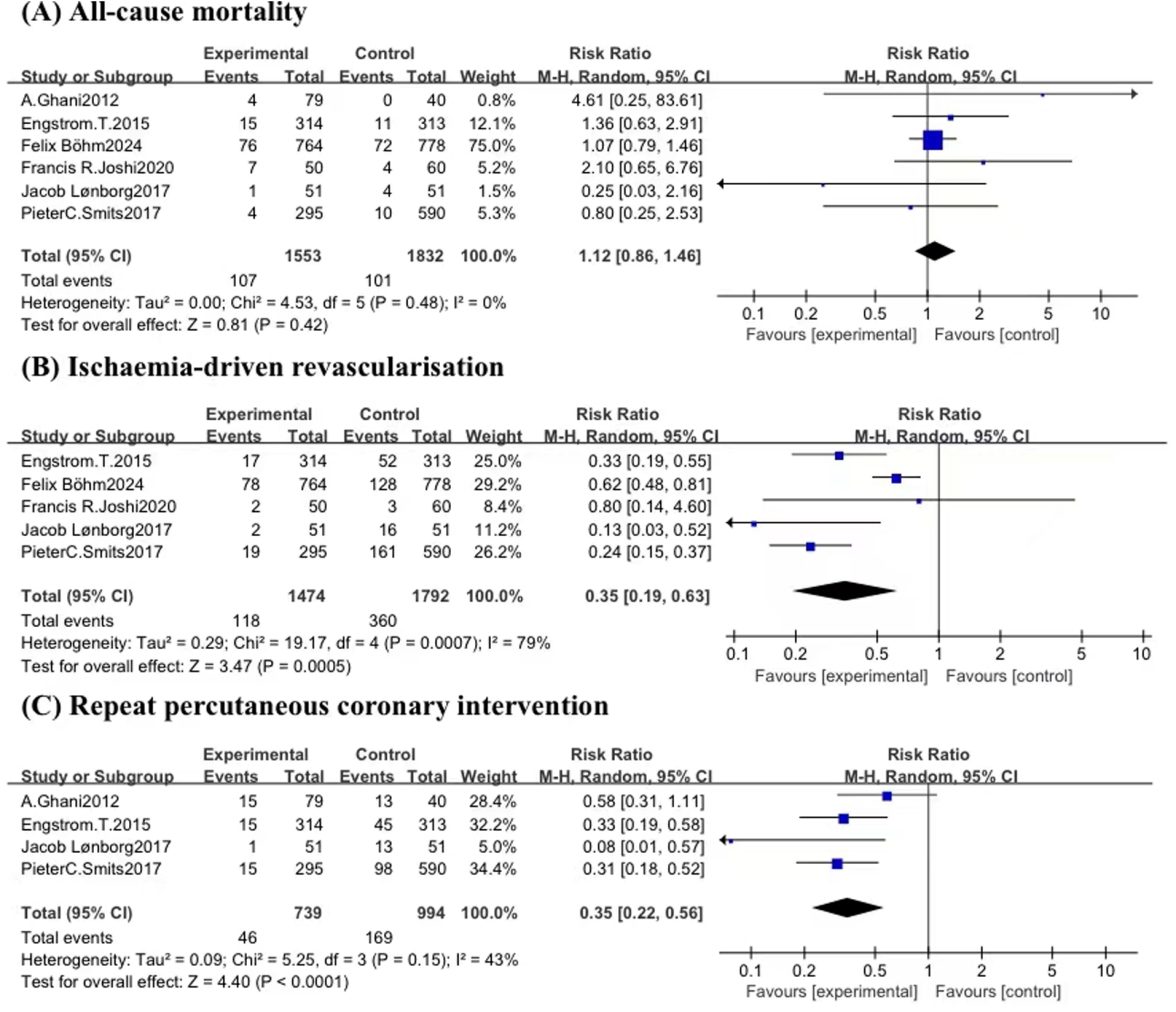
Forest plots for secondary outcomes including **(A)** all-cause mortality, **(B)** ischemia-driven revascularization, and **(C)** repeat percutaneous coronary intervention.

**Figure 6:**
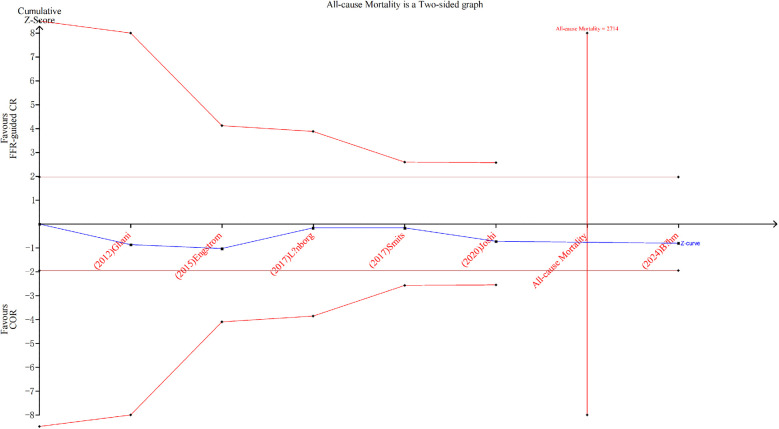
Trial sequential analysis for all-cause mortality outcome.

Among the six studies (5, 6, 9, 18–20) reporting all-cause death, 107 deaths (6.9%) among 1,553 patients occurred with FFR-guided CR vs. 101 deaths (5.5%) among 1,832 patients with COR, which did not reveal any noteworthy variance in statistical terms between the two groups(RR: 1.12, 95% CI: 0.86–1.46, 95% PI: 0.79–1.58; *P* = 0.42; *I*^2^ = 0%) (Figure 5A). Trial sequential analysis revealed that the required sample size (2,714) to reach safe conclusions was reached (Figure 6).

Five trials (6, 9, 18–20) provided data on ischemia-driven revascularization. For revascularization, a consistent benefit with CR was found compared with COR when an FFR-guided non-culprit lesion percutaneous coronary intervention strategy was used (RR: 0.35, 95% CI: 0.19–0.63; *P* = 0.00005), although there was considerable heterogeneity (*I*^2^ = 79%). A leave-one-out sensitivity analysis was conducted, revealing that heterogeneity was absent when Böhm et al.'s study (9) was excluded (Supplementary Table S6). A potential explanation for these findings is the variation in guiding strategies for revascularization. In contrast to other studies included in our analysis, this particular study focused on any planned or unplanned revascularization, which is more lenient on revascularization metrics. This difference may have contributed to the observed heterogeneity. However, when we removing this study, the results showed that FFR-guided CR led to a significant reduction in ischemia-driven revascularization (RR: 0.27, 95% CI: 0.19 −0.40, 95% PI: 0.16–0.46; *P* < 0.00001; *I*^2^ = 14%) (Figure 5B).

Repeat percutaneous coronary intervention was reported in four studies (5, 6, 19, 20). The occurrence of repeat percutaneous coronary intervention was 6.2% within FFR-guided CR group compared to 17.0% in COR group, demonstrating a marked decrease in the FFR-guided group (RR: 0.35, 95% CI: 0.22–0.56, 95% PI: 0.16–0.78; *P* < 0.0001; *I*^2^ = 43%) (Figure 5C).

Cardiac death was assessed in four trials (6, 9, 19, 20). The incidence of cardiac death showed a borderline statistical significance favoring FFR-guided CR (RR: 0.69, 95% CI: 0.48–0.99; *P* = 0.05), though the 95% PI (0.38–1.25) included 1.0, indicating uncertainty in the consistency of this effect across populations. Thus, while the point estimate suggests a potential reduction in cardiac death, the variability across studies precludes definitive conclusions. No heterogeneity was detected for this outcome measure (I² = 0%) (Supplementary Figure S2A).

Similarly, no differential association of treatment was found between FFR-guided CR strategy compared with COR strategy on repeat myocardial infarction (RR: 0.91, 95% CI: 0.58–1.42, 95% PI: 0.58–1.55; *P* = 0.68; *I*^2^ = 32%), major bleeding (RR: 1.24, 95% CI: 0.65–2.35, 95% PI: 0.28–3.27; *P* = 0.51; *I*^2^ = 0%), coronary artery bypass grafting (RR: 0.97, 95% CI: 0.30 −3.13, 95% PI: 0.04–21.13; *P* = 0.95; *I*^2^ = 48%), stroke (RR: 1.32, 95% CI: 0.46–3.82, 95% PI: 0.00–16,643.00; *P* = 0.60; *I*^2^ = 29%), contrast-induced nephropathy (RR: 0.98, 95% CI: 0.75 −1.28, 95% PI: 0.18–5.52; *P* = 0.90; *I*^2^ = 0%) and re-hospitalization (RR: 0.72, 95% CI: 0.44 −1.15, 95% PI: 0.02–33.70; *P* = 0.17; *I*^2^ = 28%) (Supplementary Figures S2B–G).

## Discussion

This meta-analysis comparing FFR-guided CR with COR in STEMI patients with multivessel disease shows that FFR-guided CR significantly reduces major adverse cardiac events, including all-cause mortality, non-fatal myocardial infarction, and ischemia-driven revascularization. The decrease in ischemia-driven revascularization parallels the reduction in major adverse cardiac events, indicating that the lower revascularization rates contribute to fewer adverse events overall. Additionally, FFR-guided CR was associated with reduced rates of repeat percutaneous coronary intervention compared to COR, with no significant increase in all-cause mortality or non-fatal myocardial infarction. FFR-guided CR showed a nominally significant reduction in cardiac death (RR: 0.69, 95% CI: 0.48–0.99; *P* = 0.05), but the wide 95% PI (0.38–1.25) suggests this benefit may not generalize uniformly. Larger trials are needed to confirm this trend. Safety outcomes such as coronary artery bypass grafting, major bleeding, stroke, contrast-induced nephropathy, and re-hospitalization showed no significant differences between the two approaches.

The observed variability across studies—reflected by the wide PI (0.20–2.19) and moderate heterogeneity (*I*^2^ = 74%)—suggests that the magnitude of benefit from FFR-guided CR may vary across clinical settings. These differences may arise from variations in patient characteristics, procedural techniques, and follow-up duration. The Ghani trial (5) was the first to utilize FFR in this context but showed no significant benefit and raised concerns about a potential increased risk of myocardial infarction. This finding was echoed by some observational studies (21, 22). Conversely, larger trials like the DANAMI 3—PRIMULTI trial (18) and the Compare-Acute trial (20) found that FFR-guided CR could reduce major adverse cardiac events and repeat revascularizations but did not affect all-cause mortality. Noteworthily, the FULL REVASC trial (9) revealed that FFR-guided CR could reduce the rate of major adverse cardiac events, although this method did not show a lower risk of all-cause mortality. These studies lacked the capacity to distinguish between the two techniques' key prognostic and safety outcomes, although they were sufficient to identify differences in the primary outcome, significant adverse cardiac events.

Critically, individual trials may lack the power to accurately assess mortality, as previous trials (5, 16, 20) have predominantly focused on evaluating major adverse cardiac events. Nonetheless, our meta-analysis combined data from these studies that met the minimum information size required in TSA, confirming that FFR-guided CR was associated with a lower rate of major adverse cardiac events and ischemia-driven revascularization without a higher rate of adverse events like repeat myocardial infarction, coronary artery bypass grafting, major bleeding, stroke, contrast-induced nephropathy, or re-hospitalization compared to COR.

Our meta-analysis aligns with previous meta-analyses (23, 24). The recent meta-analysis (25) reported that FFR-guided CR outperforms COR in terms of major adverse cardiac events and ischemia-driven repeat revascularization, with both approaches showing similar results in all-cause mortality. However, that study's small sample size precluded a thorough safety analysis of FFR-guided CR. By including newer, larger studies (9), we were able to more robustly validate that FFR-guided CR reduces the rate of major adverse cardiac events and revascularization without impacting all-cause mortality. Additionally, we demonstrated that FFR-guided CR does not significantly increase the risk of non-fatal myocardial infarction, major bleeding, myocardial infarction, or stroke. Therefore, our data support the reliability, feasibility, and safety of FFR-guided CR in patients with STEMI and multivessel disease.

Our findings should also be contextualized against angio-guided CR strategies. Prior meta-analyses of angio-guided CR in STEMI patients with multivessel disease have demonstrated reductions in cardiovascular mortality and myocardial infarction compared to COR. For instance, a pooled analysis by Bainey et al. (26) reported a 26% reduction in cardiovascular mortality (RR: 0.74, 95% CI: 0.58–0.95) and a 32% reduction in recurrent myocardial infarction (RR: 0.68, 95% CI: 0.50–0.92) with angio-guided CR. In contrast, our meta-analysis of FFR-guided CR showed no significant reduction in all-cause mortality (RR: 1.12, 95% CI: 0.86–1.46) or myocardial infarction (RR: 0.91, 95% CI: 0.58–1.42), despite similar reductions in ischemia-driven revascularization. This discrepancy may reflect differences in lesion selection: FFR guidance avoids unnecessary interventions in functionally non-significant lesions, potentially mitigating procedural risks but limiting the opportunity to stabilize high-risk plaques that angiographically appear severe (27). Conversely, angio-guided CR may inadvertently treat lesions with lower ischemic burden, yet its broader intervention scope might address vulnerable plaques, thereby reducing myocardial infarction and mortality (28). Future trials directly comparing FFR- and angio-guided CR are warranted to elucidate these mechanistic differences.

### Limitations

This meta-analysis had certain drawbacks. First, the scheduling of the FFR measurement differed among the trials. In the Compare-Acute investigation (18) and the research by Joshi et al. (18), FFR was executed concurrently with the initial percutaneous coronary intervention. Conversely, in the DANAMI 3-PRIMULTI trial (6) and Lønborg et al.'s examination (19), the FFR evaluation was carried out two days post the primary procedure. The time of the FFR measurement was not strictly regulated in the following two trials (5, 9); it could be done at the operator's discretion either during the index surgery or later during the index hospitalization. Therefore, research on the instantaneous wave-free ratio (iFR) in this context is warranted (29). Secondly, different eras might have different techniques (like evolving interventional procedures), devices (like stent types), concepts, and the introduction of new drugs, which could affect the results. Thirdly, the cut-off value of FFR was different, the Ghani trial (5) used 0.75, while the rest used 0.80. Finally, since all included studies were conducted in developed countries, our findings are only applicable to those regions, and further research in other countries is needed to enhance generalizability.

### Implications of these findings in practice

Our findings suggest that FFR-guided CR reduces major adverse cardiac events in multivessel revascularization for STEMI patients. Physicians should consider patient-specific factors and monitor kidney function, given the potential for complications such as major bleeding and stroke with both FFR-guided CR and COR. Due to the higher complication risks in these patients (30), careful monitoring during and after FFR-guided revascularization is recommended. For patients with borderline lesions, FFR measurements may help guide treatment strategies based on lesion functional significance.

### Implications of these findings in future research

We suggest that trials for chronic complete occlusions be carried out in the future to determine the actual impact of FFR-guided percutaneous coronary intervention on distal and multivessel revascularization. FFR-guided percutaneous coronary intervention may still be clinically beneficial for patients with coronary artery disease, according to a single-center retrospective investigation alone (31). As a result, nothing is known about how FFR-guided percutaneous coronary intervention actually works in individuals who have persistent complete occlusions. Since stroke and cerebrovascular events were recorded in relatively few trials, we therefore suggest including them as outcomes of interest. Consequently, we were unable to look at how FFR-guided CR affected the progression of cerebrovascular events. We suggest more research to support this claim. A network indirect meta-analysis could provide valuable insights by enabling a comprehensive comparison of FFR-guided CR, COR, and standard CR. However, despite conducting an extensive literature search, the number of available randomized controlled trials was insufficient to perform a robust network meta-analysis (Two for FFR-guided CR VS conservative treatment (32, 33); six for COR VS CR (21, 34–38). This limitation underscores the need for further high-quality studies directly comparing these revascularization strategies to guide clinical practice.

## Conclusion

Our meta-analysis demonstrates that FFR-guided CR is associated with a reduced risk of major adverse cardiac events compared to COR, supporting its beneficial role in improving cardiac outcomes. However, the observed variability across studies suggests that the magnitude of this benefit may not be uniformly consistent across all clinical settings. While these findings highlight the potential clinical value of FFR-guided CR, further research is needed to confirm its consistency and to better define its applicability in diverse patient populations and healthcare environments.

## Data Availability

The original contributions presented in the study are included in the article/Supplementary Material, further inquiries can be directed to the corresponding author.
