# Supplementary material for: Fractional flow reserve-guided complete vs. culprit-only revascularization in ST-elevation myocardial infarction patients with multivessel disease: a meta-analysis
Source: Front Cardiovasc Med. 2025 Mar 11;12:1509912. doi: 10.3389/fcvm.2025.1509912 (PMC11933064; doi:10.3389/fcvm.2025.1509912)
Supplement: Supplementary file 1 [file Datasheet1.doc]

**Supplementary Material**

**STable 1** Full search strategies used for PubMed, Cochrane Library, Embase and CNKI.

**STable 2** Summary of six RCTs comparing complete revascularization with culprit-lesion-only PCI.

**STable 3** The Clinical outcomes extracted from the six articles.

**STable 4** Grading of Recommendations, Assessment, Development, and Evaluations (GRADE).

**STable 5** Leave-one-out sensitivity analysis for major adverse cardiac events.

**STable 6** Leave-one-out sensitivity analysis for ischemia-driven revascularization.

**SFig. 1** Trial sequential analysis for major adverse cardiac events.

**SFig. 2** Forest plots for secondary outcomes including (A) cardiac death, (B) repeat myocardial infarction, (C) major bleeding, (D) coronary artery bypass graft, (E) stroke, (F) contrast-induced nephropathy and (G) Rehospitalization.

**SFig. 3** Funnel Plot for secondary outcomes including (A) cardiac death, (B) repeat myocardial infarction, (C) major bleeding, (D) coronary artery bypass graft, (E) stroke, (F) contrast-induced nephropathy and (G) Rehospitalization.

**SFig. 4** Leave-one-out sensitivity analysis for (A) major adverse cardiac event, (B) all-cause mortality, (C) ischemia-driven revascularization, (D) repeat percutaneous coronary intervention (Stata 15.0).

**SFig. 5** Leave-one-out sensitivity analysis for (A) cardiac death, (B) repeat myocardial infarction, (C) major bleeding, (D) coronary artery bypass graft, (E) stroke, (F) contrast-induced nephropathy and (G) Rehospitalization (Stata 15.0).

**STable 1** Full search strategies used for PubMed, Embase, Cochrane Library and CNKI.

| **PubMed** | | |
| --- | --- | --- |
|  | "Myocardial Infarction"[Mesh] | 219566 |
|  | "Culprit-Only PCI"[Mesh] | 62 |
|  | (((((((Myocardial Infarct[Title/Abstract]) OR (Heart Attack[Title/Abstract])) OR (acute coronary syndrome[Title/Abstract])) OR (ST-elevation myocardial infarction[Title/Abstract])) OR (ST-segment elevation myocardial infarction[Title/Abstract])) OR (ST-elevation[Title/Abstract])) OR (STEMI[Title/Abstract])) OR (MI[Title/Abstract]) | 139969 |
|  | ((((((((((percutaneous coronary intervention[Title/Abstract]) OR (primary perctaneous coronary intervention[Title/Abstract])) OR (revascularization[Title/Abstract])) OR (angioplasty[Title/Abstract])) OR (PCI[Title/Abstract])) OR (culprit vessel revascularization[Title/Abstract])) OR (culprit vessel percutaneous coronary intervention[Title/Abstract])) OR (target vessel revascularization[Title/Abstract])) OR (culprit coronary artery revascularization[Title/Abstract])) OR (culprit-only revascularization[Title/Abstract])) OR (culprit lesion[Title/Abstract]) | 148743 |
|  | FFR-guided complete revascularization[Title/Abstract] | 11 |
|  | FFR[Title/Abstract] | 4556 |
|  | complete revascularization[Title/Abstract] | 1739 |
|  | non-culprit lesion[Title/Abstract] | 86 |
|  | multivessel percutaneous coronary intervention[Title/Abstract] | 170 |
|  | #1 OR #3 | 259772 |
|  | #2 OR #4 | 148743 |
|  | #5 OR #6 OR #7 OR #8OR#9 | 6460 |
|  | (((randomized controlled trial[Title/Abstract]) OR (controlled clinical trial[Title/Abstract])) OR (randomized[Title/Abstract])) OR (randomly trial[Title/Abstract]) | 749673 |
|  | #10AND #11AND #12 AND #13 | 331 |
| **COCHRANE CENTRAL** | | |
| 1. | MeSH descriptor: [Myocardial Infarction] explode all trees | 15687 |
| 2. | MeSH descriptor: [Percutaneous Coronary Intervention] explode all trees | 8867 |
| 3. | MeSH descriptor: [Randomized Controlled Trial] explode all trees | 37 |
| 4. | (Myocardial Infarction) | 39087 |
| 5. | (percutaneous coronary intervention) | 14319 |
| 6. | (randomized controlled trial) | 1437998 |
| 7. | #1 OR #4 | 39226 |
| 8. | #2 OR #5 | 16664 |
| 9. | #3 OR #6 | 1437998 |
| 10. | #7 AND #8 AND #9 | 8476 |
| 11. | (FFR-guided complete revascularization) | 50 |
| 12. | (FFR) | 700 |
| 13. | (complete revascularization) | 1914 |
| 14 | (Multi-vessel disease) | 307 |
| 15 | (non-culprit lesion) | 165 |
| 16 | (multivessel percutaneous coronary intervention) | 824 |
| 17 | #11OR#12OR#13OR#14OR#15OR#16 | 3300 |
| 18 | #10AND#17 | 988 |
| **EMBASE** | | |
| 1. | 'heart infarction'/exp | 488977 |
| 2. | 'cardiac infarct':ti,ab,kw OR 'cardiac infarction':ti,ab,kw OR 'cardial infarct':ti,ab,kw OR 'heart attack':ti,ab,kw OR 'heart infarct':ti,ab,kw OR 'heart micro infarction':ti,ab,kw OR 'heart muscle infarction':ti,ab,kw OR 'infarction, heart':ti,ab,kw OR 'myocardial infarct':ti,ab,kw OR 'myocardial infarction':ti,ab,kw OR 'myocardium infarct':ti,ab,kw OR 'myocardium infarction':ti,ab,kw OR 'premonitory infarction sign':ti,ab,kw OR 'second heart attack':ti,ab,kw OR 'subendocardial infarction':ti,ab,kw OR 'transmural cardiac infarction':ti,ab,kw OR 'transmural heart infarction':ti,ab,kw OR 'transmural infarction, heart':ti,ab,kw OR 'heart infarction':ti,ab,kw | 313122 |
| 3. | #1 OR #2 | 529965 |
| 4. | 'percutaneous coronary intervention'/exp | 132871 |
| 5. | 'percutaneous coronary intervention':ti,ab,kw | 73170 |
| 6. | #4 OR #5 | 132871 |
| 7. | 'complete revascularization'/exp | 2629 |
| 8. | (('electret mask':ti,ab,kw OR 'electret respirator':ti,ab,kw OR 'ffp mask':ti,ab,kw OR 'ffp respirator':ti,ab,kw OR ffr:ti,ab,kw) AND 'filtering facepiece respirator':ti,ab,kw OR ffrs:ti,ab,kw) AND 'filtering facepiece respirators':ti,ab,kw OR 'filter mask':ti,ab,kw OR 'filtering face-piece mask':ti,ab,kw OR 'filtering face-piece respirator':ti,ab,kw OR 'filtering facepiece half mask':ti,ab,kw OR 'filtering facepiece half mask':ti,ab,kw OR 'filtering facepiece mask':ti,ab,kw OR 'filtering facepiece particulate respirator':ti,ab,kw OR 'filtering respiratory mask':ti,ab,kw OR 'particle filtering half-mask':ti,ab,kw OR 'particle filtering half mask':ti,ab,kw OR 'particle-filtering face mask':ti,ab,kw OR 'particulate air filter respirator':ti,ab,kw OR 'particulate filter respirator':ti,ab,kw OR 'particulate filtering face piece respirator':ti,ab,kw OR 'particulate filtering respirator':ti,ab,kw OR 'particulate-filtering respirator':ti,ab,kw OR 'respiratory protection mask':ti,ab,kw OR 'filtering facepiece respirator':ti,ab,kw | 378 |
| 9. | #7 OR #8 | 3007 |
| 10. | 'controlled trial, randomized':ti,ab,kw OR 'randomised controlled study':ti,ab,kw OR 'randomised controlled trial':ti,ab,kw OR 'randomized controlled study':ti,ab,kw OR 'trial, randomized controlled':ti,ab,kw OR 'randomized controlled trial':ti,ab,kw | 221872 |
| 11. | #3 AND #6 AND #9 AND #10 | 13 |

| **CNKI** | | |
| --- | --- | --- |
| 1. | Myocardial Infarction | 188949 |
| 2. | **Culprit-Only PCI** | 70 |
| 3. | **‘Myocardial Infarct’ + ’Heart Attack’ + ‘acute coronary syndrome’ + ‘ST-elevation myocardial infarction’ + ‘ST-segment elevation myocardial infarction’ + ‘ST-elevation’ + ‘STEMI’ + ‘MI’** |   105390 |
| 4. | **‘percutaneous coronary intervention’ + ‘primary perctaneous coronary intervention’ + ‘revascularization’ + ‘angioplasty’ + ‘PCI’ + ‘culprit vessel revascularization’ + ‘culprit vessel percutaneous coronary intervention’ + ‘target vessel revascularization’ + ‘culprit coronary artery revascularization’ + ‘culprit-only revascularization’** | 80021 |
| 5. | **FFR-guided complete revascularization** | 20 |
| 6. | **FFR** | 4,957 |
| 7. | **complete revascularization** | 2,076 |
| 8. | **non-culprit lesion** | 205 |
| 9. | **multivessel percutaneous coronary intervention** | 713 |
| 10. | #1 OR #3 | 201,588 |
| 11. | #2 OR #4 | 57,554 |
| 12. | #5 OR #6 OR #7 OR #8 OR #9 | 7,752 |
| 13. | **‘randomized controlled trial’ + ‘controlled clinical trial’ + ‘randomized’ + ‘randomly trial’** | 1,657 |
| 14. | #10 AND #11 AND #12 AND #13 | 483 |

**STable 2** Summary of six randomized controlled studies comparing FFR-guided complete revascularization with culprit-only revascularization.

| **Source** | **Intervention** | **Experimental group** | **Control group** | **Indication of non-IRA intervention** | **Primary**  **Outcome** | **Secondary endpoint** | **Stent type** |
| --- | --- | --- | --- | --- | --- | --- | --- |
| **Ghani^5^**  **2012** | FFR-guided PCI in vessels with significant stenosis (<90%) vs cuipirit-vessel PCI plus PCI of several lesion (>90%) | 80 | 41 | FFR < 0.75 Or Diameter stenosis > 90% | MACE | Death, Re-PCI, Re-PCI NCL, Re-PCI CL,CABG, Re-MI, Death and/or MI. | DES 20%  BMS 68% |
| **Engstrøm^6^**  **2015** | revascularisation guided by FFR values vs no further invasive treatment after primary PCI of the infarct-related artery only | 314 | 313 | FFR<0.80 Or Diameter stenosis > 50% | MACE | Cardiac death, cardiac death or non-fatal, MI, urgent PCI, Non-urgent PCI, unplanned CABG, periprocedural MI, bleeding, contrast-induced nephropathy, stroke. | DES 94% BMS 1% |
| **Böhm^9^**  **2024** | FFR-guided complete revascularization vs culprit-lesion-only PCI | 764 | 778 | FFR < 0.80 Or Diameter stenosis between 90-99% | MACE | Death from any cause or MI, unplanned revascularization, death from any cause, death from cardiovascular causes, MI, any planned or unplanned and revascularization, cardiovascular death-myocardial infarction-or unplanned revascularization, any stent thrombosis, any restenosis, target vessel revascularization, stroke, major bleeding, re-hospitalization. | DES 95% |
| **Joshi^18^**  **2020** | FFR-guided complete revascularization vs infarct-related (culprit) artery-only PCI | 50 | 60 | FFR < 0.80 Or Diameter stenosis > 50% | MACE | All-cause death, MI and ischaemia-driven revascularization. | DES 2% |
| **Lønborg^19^**  **2017** | FFR–guided complete revascularization vs infarct-related percutaneous coronary intervention only. | 97 | 100 | FFR < 0.80 Or Diameter stenosis ≥ 90%. | All-cause mortality, nonfatal reinfarction and ischemia-driven revascularization | Cardiac death, all-cause mortality or nonfatal reinfarction, urgent PCI, non-urgent PCI, unplanned CABG and any target vessel revascularization | DES 94% |
| **Smits^20^**  **2017** | non–infarct-related coronary arteries guided by FFR vs culprit-lesion-only PCI | 295 | 590 | FFR < 0.80 Or Angiographic stenosis ≥ 50% | MACCE | NACE (any first event), death from any cause) or myocardial Infarction, major bleeding, hospitalization any revascularization and stent thrombosis | DES 97% |

Abbreviations: CL, culprit lesion; MACE, major adverse cardiac events; MACCE, denotes the composite of MACE and cerebrovascular events; MI, myocardial infarction; NCL, non-culprit lesion; PCI, percutaneous coronary intervention; CABG, coronary artery bypass graft; NR ,not report; DES, drug-eluting stent; BMS, bare mental stent.

STable 3 The Clinical outcomes extracted from the six randomized controlled studies.

| **Study** | **Patient** | **Primary Outcome** | **Secondary Outcomes** | | | | | | | | | |
| --- | --- | --- | --- | --- | --- | --- | --- | --- | --- | --- | --- | --- |
|  |  | MACE | All-cause mortality | Ischaemia-driven revascularisation | Re-PCI | Cardiac death | Re-MI | Major Bleeding* | CABG | Stroke | Contrast-induced nephropathy# | Re-hospitalization |
| **Ghani^5^** | 79/40 | 28/14 | 4/0 | NR | 15/13 | NR | 14/0 | NR | 12/1 | NR | NR | NR |
| **Engstrøm^6^** | 314/313 | 40/68 | 15/11 | 17/52 | 15/45 | 5/9 | 15/16 | 1/4 | 3/7 | 4/1 | 6/7 | NR |
| **Böhm^9^** | 764/778 | 104/132 | 76/72 | 78/128 | NR | 32/40 | 61/58 | 19/17 | NR | 22/22 | 89/91 | 23/26 |
| **Joshi^18^** | 50/60 | NR | 7/4 | 2/3 | NR | NR | 2/2 | NR | NR | NR | NR | NR |
| **Lønborg^19^** | 51/51 | NR | 1/4 | 2/16 | 1/13 | 1/3 | 2/4 | NR | 1/3 | NR | NR | NR |
| **Smits^20^** | 295/590 | 23/117 | 4/10 | 19/161 | 15/98 | 7/28 | 7/28 | 3/8 | 3/5 | NR | NR | 13/47 |

Values are reported as experimental group/ Control group. Abbreviations: MACE, major adverse cardiac events; Re-MI, repeat myocardial infarction; Re-PCI, repeat percutaneous coronary intervention; CABG, coronary-artery bypass graft surgery.

* Major Bleeding, requiring transfusion or surgery.

\# Contrast-induced nephropathy, >50% rise in plasma creatinine.

**STable 4** Grading of Recommendations, Assessment, Development, and Evaluations (GRADE).

| **Quality assessment** | | | | | | | **No of patients** | | **Effect** | | **Quality** | **Importance** |
| --- | --- | --- | --- | --- | --- | --- | --- | --- | --- | --- | --- | --- |
|  |  |  |  |  |  |  |  |  |  |  |  |  |
| **No of studies** | **Design** | **Risk of bias** | **Inconsistency** | **Indirectness** | **Imprecision** | **Other considerations** | **FFR-CR** | **COR** | **RR (95% CI)** | **Absolute** |  |  |
| **All-cause mortality** | | | | | | | | | | | | |
| 6 | randomised trials | no serious risk of bias | no serious inconsistency | no serious indirectness | serious^1^ | none | 107/1553  (6.9%) | 101/1832  (5.5%) | 1.12  (0.86-1.46) | 7 more per 1000  (8 fewer to 25 more) | ⊕⊕⊕O MODERATE | CRITICAL |
| **Major adverse cardiac events** | | | | | | | | | | | | |
| 4 | randomised trials | no serious risk of bias | no serious inconsistency | no serious indirectness | no serious imprecision | none | 195/1452  (13.4%) | 331/1721  (19.2%) | 0.66  (0.46-0.94) | 65 fewer per 1000  (12 fewer to 104 fewer) | ⊕⊕⊕⊕ HIGH | CRITICAL |
| **Ischemia-driven revascularization** | | | | | | | | | | | | |
| 4 | randomised trials | serious^2^ | no serious inconsistency | no serious indirectness | no serious imprecision | none | 40/710  (5.6%) | 232/1014  (22.9%) | 0.27  (0.19-0.4) | 167 fewer per 1000  (137 fewer to 185 fewer) | ⊕⊕⊕O MODERATE | CRITICAL |
| **Repeat percutaneous coronary intervention** | | | | | | | | | | | | |
| 4 | randomised trials | no serious risk of bias | no serious inconsistency | no serious indirectness | no serious imprecision | none | 46/739  (6.2%) | 169/994  (17%) | 0.35  (0.22-0.56) | 111 fewer per 1000  (75 fewer to 133 fewer) | ⊕⊕⊕⊕ HIGH | CRITICAL |
| **Cardiac death** | | | | | | | | | | | | |
| 4 | randomised trials | no serious risk of bias | serious^2^ | no serious indirectness | no serious imprecision | none | 45/1424  (3.2%) | 80/1732  (4.6%) | 0.69  (0.48-0.99) | 14 fewer per 1000  ( 0 fewer to 24 fewer) | ⊕⊕⊕O MODERATE | IMPORTANT |
| **Repeat myocardial infarction** | | | | | | | | | | | | |
| 6 | randomised trials | no serious risk of bias | no serious inconsistency | no serious indirectness | no serious imprecision | none | 101/1553  (6.5%) | 108/1832  (5.9%) | 0.91  (0.58-1.42) | 5 fewer per 1000  (25 fewer to 25 more) | ⊕⊕⊕⊕ HIGH | IMPORTANT |
| **Major bleeding** | | | | | | | | | | | | |
| 3 | randomised trials | no serious risk of bias | no serious inconsistency | no serious indirectness | no serious imprecision | none | 23/1373  (1.7%) | 29/1681  (1.7%) | 0.95  (0.54-1.67) | 1 fewer per 1000  (8 fewer to 12 more) | ⊕⊕⊕⊕ HIGH | IMPORTANT |
| **Coronary artery bypass graft** | | | | | | | | | | | | |
| 4 | randomised trials | no serious risk of bias | no serious inconsistency | no serious indirectness | no serious imprecision | none | 19/739  (2.6%) | 16/994  (1.6%) | 0.97  (0.3-3.13) | 0 fewer per 1000  (11 fewer to 34 more) | ⊕⊕⊕⊕ HIGH | IMPORTANT |
| **Stroke** | | | | | | | | | | | | |
| 2 | randomised trials | serious^2^ | no serious inconsistency | no serious indirectness | serious^1^ | none | 26/1078  (2.4%) | 23/1091  (2.1%) | 1.32  (0.46-3.82) | 7 more per 1000  (11 fewer to 59 more) | ⊕⊕OO LOW | IMPORTANT |
| **Contrast-induced nephropathy** | | | | | | | | | | | | |
| 2 | randomised trials | no serious risk of bias | no serious inconsistency | no serious indirectness | no serious imprecision | none | 95/1075  (8.8%) | 98/1085  (9%) | 0.98  (0.75-1.28) | 2 fewer per 1000  (23 fewer to 25 more) | ⊕⊕⊕⊕ HIGH | IMPORTANT |
| **Re-hospitalization** | | | | | | | | | | | | |
| 2 | randomised trials | no serious risk of bias | no serious inconsistency | no serious indirectness | no serious imprecision | none | 36/1059  (3.4%) | 73/1368  (5.3%) | 0.72 (0.44-1.15) | 15 fewer per 1000  (30 fewer to 8 more) | ⊕⊕⊕⊕ HIGH | IMPORTANT |

1 Trusted interval width

2 Random, allocation of hidden and blind methods are biased.

STable 5 Leave-one-out sensitivity analysis for major adverse cardiac events

|  | **RR** | **95% CI** | **P-value** | **Tau^2^** | **Chi^2^** | **I^2^** |
| --- | --- | --- | --- | --- | --- | --- |
| **Omitting Ghani, 2012** [5] | 0.58 | [0.38; 0.89] | 0.01 | 0.11 | 9.65 | 79% |
| **Omitting Engstrom, 2015** [6] | 0.67 | [0.40; 1.15] | 0.15 | 0.18 | 11.54 | 83% |
| **Omitting Böhm, 2024** [9] | 0.60 | [0.36; 1,00] | 0.05 | 0.16 | 8.63 | 77% |
| **Omitting Smits, 2017** [20] | 0.76 | [0.58; 0.99] | 0.04 | 0.02 | 3.42 | 41% |

Abbreviations: RR, relative risk; CI, confidence interval

STable 6 Leave-one-out sensitivity analysis for ischemia-driven revascularization

|  | **RR** | **95% CI** | **P-value** | **Tau^2^** | **Chi^2^** | **I^2^** |
| --- | --- | --- | --- | --- | --- | --- |
| **Omitting Engstrom, 2015 [6]** | 0.35 | [0.16; 0.79] | 0.01 | 0.45 | 17.63 | 83% |
| **Omitting Böhm, 2024 [9]** | 0.27 | [0.19; 0.40] | < 0.00001 | 0.02 | 3.49 | 14% |
| **Omitting Joshi, 2020 [18]** | 0.32 | [0.17; 0.61] | 0.0005 | 0.32 | 18.74 | 84% |
| **Omitting Lønborg, 2017 [19]** | 0.40 | [0.22; 0.73] | 0.003 | 0.26 | `15.82 | 81% |
| **Omitting Smits, 2017 [20]** | 0.41 | [0.22; 0.76] | 0.005 | 0.22 | 9.02 | 67% |

Abbreviations: RR, relative risk; CI, confidence interval

**SFig. 1** Trial sequential analysis for major adverse cardiac events.

**SFig. 2** Forest plots for safety outcomes including (A) Cardiac death, (B) Repeat myocardial infarction, (C) Major bleeding, (D) Coronary artery bypass graft, (E) Stroke, (F) Contrast-induced nephropathy and (G) Rehospitalization.

**SFig. 3** Funnel Plot for secondary outcomes including (A) cardiac death, (B) repeat myocardial infarction, (C) major bleeding, (D) coronary artery bypass graft, (E) stroke, (F) contrast-induced nephropathy and (H) Rehospitalization.

**SFig. 4** Leave-one-out sensitivity analysis for (A) major adverse cardiac event, (B) all-cause mortality, (C) ischemia-driven revascularization, (D) repeat percutaneous coronary intervention (Stata 15.0).

**SFig. 5** Leave-one-out sensitivity analysis for (A) cardiac death, (B) repeat myocardial infarction, (C) major bleeding, (D) coronary artery bypass graft, (E) stroke, (F) contrast-induced nephropathy and (H) Rehospitalization (Stata 15.0).
